# Fostering autonomous motivation, physical activity and cardiorespiratory fitness in rheumatoid arthritis: protocol and rationale for a randomised control trial

**DOI:** 10.1186/1471-2474-15-445

**Published:** 2014-12-19

**Authors:** Peter C Rouse, Jet JCS Veldhuijzen Van Zanten, George S Metsios, Nikos Ntoumanis, Chen-an Yu, Yiannis Koutedakis, Sally AM Fenton, Joanna Coast, Hema Mistry, George D Kitas, Joan L Duda

**Affiliations:** Department of Health, University of Bath, Bath, BA2 7AY UK; School of Sport, Exercise and Rehabilitation Sciences, University of Birmingham, Edgbaston, Birmingham B15 2TT UK; Institute of Sport, Faculty of Health, Education and WellBeing, University of Wolverhampton, Wolverhampton, WV1 1LY UK; Department of Rheumatology, The Dudley Group NHS Foundation Trust, Russell’s hall Hospital, Dudley, DY1 2HQ UK; School of Psychology and Speech Pathology, Curtin University, Perth, 6845 Western Australia; School of Health and Population Sciences, University of Birmingham, Birmingham, B15 2TT UK; Warwick Medical School, University of Warwick, Coventry, CV4 7AL UK

**Keywords:** Rheumatoid arthritis, Physical activity, Behaviour change, Self-determination theory, Autonomy support, Autonomous motivation

## Abstract

**Background:**

People with rheumatoid arthritis are at greater risk of morbidity and mortality from cardiovascular disease than the general population. Sustained physical activity increases cardio-respiratory fitness and reduces cardiovascular disease risk factors. However, little is known about how we can effectively promote long-term participation in physical activity in patients with rheumatoid arthritis. The literature consistently calls for physical activity interventions, and their implementation, to be theoretically-grounded.

**Methods/Design:**

This paper documents the protocol of a randomised control trial that investigates whether a Self-determination Theory-based intervention fosters the adoption and maintenance of physical activity (3, 6 and 12 months) sufficient to provide sustained cardiovascular and personal well-being benefits in patients with rheumatoid arthritis. The cost effectiveness of the intervention will also be determined. The trial is registered as Current Controlled Trials ISRCTN04121489.

**Discussion:**

Results from this trial will provide guidance regarding key social environmental factors that can be manipulated to support motivational processes conducive to positive health behaviour change and optimal functioning in patients with Rheumatoid Arthritis.

**Electronic supplementary material:**

The online version of this article (doi:10.1186/1471-2474-15-445) contains supplementary material, which is available to authorized users.

## Background

Rheumatoid arthritis (RA) is the most common chronic inflammatory arthritis affecting ~1% of British adults [1], it causes joint pain, stiffness, swelling and can eventually lead to structural damage and physical dysfunction. Consequently, people with RA commonly experience psychological distress, particularly heightened anxiety, depression and life dissatisfaction, accompanied with feelings of reduced personal autonomy and functional independence [2–4]. In addition, RA is associated with increased morbidity and mortality, particularly from cardiovascular disease (CVD) [5, 6], with both myocardial infarction and stroke being more prevalent than in the general population [7, 8]. The increased risk of CVD is attributed to both classical (e.g. hypertension, dyslipidaemia) [9, 10] and novel or disease-related factors (e.g., inflammation) [11]. Regular physical activity (PA) of sufficient intensity increases cardio-respiratory fitness and has been repeatedly shown to reduce individual CVD risk factors as well as overall CVD risk in both healthy [12] and diseased populations such as cardiac and diabetic patients [13]. Evidence indicates that exercise interventions in patients with RA lead to reduced feelings of fatigue, improved cardio-respiratory fitness, physical function and psychological well-being, without aggravating symptoms or inducing further joint damage [14, 15]. More recently, regular exercise was also shown to improve the cardiovascular risk profile in patients with RA [16, 17].

### Physical activity and rheumatoid arthritis

Even though RA patients are aware of the benefits of PA [18, 19], the majority of the patients are not physically active [20, 21]. However, regular PA is a realistic and important component to a holistic treatment programme for this patient group. As mentioned above, a recent study revealed that an individualised exercise intervention induced reductions in CVD risk in patients with RA [12]. Patients who received the exercise intervention showed reductions in blood pressure, lipid ratio and inflammation, as well as increases in fitness and functional ability, compared to an advice-only control group. In other patient groups (e.g., hypertensive and overweight patients), exercise training programmes of several weeks or months in duration have enhanced insulin sensitivity [22], reduced blood pressure [23], improved lipoprotein profile and decreased body fatness [24], and may even have an anti-inflammatory effect [25]. These data clearly suggest that most CVD risk factors, which are particularly pronounced in RA patients, can be beneficially modified by increasing levels of PA engagement [14, 26]. Still, whilst such exercise programmes have demonstrated short-term improvements in patient health [27], there is no compelling evidence for sustained participation in PA post programmes and associated long-term improvements in specific outcomes. Indeed, the possible cardiovascular benefits of exercise only persist with continued and long-term PA participation. Understanding the processes linked to adherence to individualised exercise protocols after cessation of structured programmes is therefore central to the success of PA promotion programmes which seek to bring about lasting health benefits. The mechanisms underlying maintenance of PA engagement have not been studied in RA patients [14]. Therefore, research is needed to understand how to optimally support long-term PA participation in this patient group.

This paper documents the rationale for, and protocol of a randomised control trial (RCT) that compares two 3-month exercise programmes, with the primary aim of improving cardio-respiratory fitness among patients with RA. The exercise component of both programmes is the same, but one programme is supplemented by a theoretically-grounded behaviour change intervention. Specifically, this intervention aimed to target the key motivational processes underlying PA behaviour change, with the intention of encouraging the adoption and maintenance of PA and in turn, improving cardio-respiratory fitness among RA patients.

### Theoretical framework

In order to take into account key underlying processes relevant to PA adherence and optimal functioning, interventions should be theoretically based [27, 28]. Systematic and meta-analytic reviews [29, 30] indicate that Self-determination Theory (SDT; [31]) holds promise for understanding the processes that lead to sustained health behaviour change and well-being. SDT is concerned with the determinants and implications of ‘why’ we engage in specific behaviours. Specifically, SDT focuses on the degree to which people’s motivation toward engagement in activities, such as PA, emanates from the self (i.e., is self-determined) or is driven by external or internal pressures. SDT proposes that when an activity is not intrinsically motivating, behaviour is guided by a variety of extrinsic regulations which are assumed to lie on a self-determination continuum, ranging from those that are more self-determined (or autonomous) to those that are less self-determined (or controlled) [32]. Research grounded in SDT [33], has highlighted the positive influence that autonomy support (e.g., eliciting and acknowledging perspectives, supporting self-initiative, offering choice, providing relevant information and minimizing pressure and control) can have on facilitating more autonomous motivation and health behaviour change, as well as associated physical and psychological health benefits [34].

To date, within the SDT-based literature centred on PA promotion, emphasis has been placed on the degree of autonomy support offered by a variety of exercise professionals (e.g. instructors and health fitness advisors [35–38]. According to SDT, the degree to which individuals experience well-being and are more autonomous in their motivation is influenced by the extent to which their innate psychological needs to feel competent, autonomous, and connected with others (sense of relatedness) are satisfied in a particular context or activity. SDT proposes that autonomy supportive interactions with significant others (e.g., exercise instructors, behavioural change counsellors) contribute to greater satisfaction of the 3 psychological needs of competence, autonomy, and relatedness, and in turn, enhanced autonomous motivation towards PA.

SDT-based investigations focussed on health behaviour change [including recent RCTs [39, 40] have shown that more self-determined regulations can predict adherence to medical prescriptions [41], smoking cessation [34], weight loss [42], and glycemic control [43]. Research particularly targeting PA has revealed positive associations between autonomy support and need satisfaction and autonomous reasons for engaging in exercise [36, 44]. For example, previous studies have shown that more autonomous motives for exercise correspond to positive outcomes such as adherence [44] and enhanced well-being [35, 44–46]. A cluster randomised control trial comparing a standard exercise referral scheme with an exercise referral intervention grounded in SDT, revealed that more health and fitness advisor autonomy support corresponded to greater self-determined motivation over the course of the 12 week programme. Greater self-determined motivation corresponded to enhanced well-being and PA engagement at six months follow-up [35]. In addition, a longitudinal study of overweight/obese individuals involved in an Exercise on Prescription programme [36], demonstrated an increase in psychological need satisfaction over time corresponded to greater adherence to the 3-month exercise prescription and more autonomous motives for PA engagement. The latter was associated with greater well-being throughout the programme. With regards to RA patients, a recent study indicated that more autonomous motivation towards PA was significantly positively associated with higher levels of self-reported PA [47].

In clinical populations, PA promotion is primarily carried out via supervised, hospital based exercise programmes. However, RA patients are often inappropriately excluded from exercise programmes, which are known to reduce the risk of CVD in the general population. The present multi-component intervention is based on psychological theory as well as physiological principles of safe and appropriate exercise programmes for patients with RA [14]. In developing the intervention package, we assumed that the goals of maintained PA behaviour change and related positive health benefits necessitate the employment of different but complementary intervention strategies [27]. As such, this trial reflects an interdisciplinary collaboration between researchers with expertise in behavioural and motivation psychology, exercise physiology and rheumatology.

### Aims

The present RCT aims first, to investigate whether a SDT-based psychological intervention plus exercise programme customised for patients with RA, fosters the adoption and maintenance of PA (3, 6 and 12 months) sufficient to improve VO_2_ max and sustain cardiovascular and personal well-being benefits in patients with RA, compared to a standard provision exercise programme customised for this particular patient group;. and second, whether this intervention is cost-effective relative to an exercise programme alone. Specifically, this RCT examined the effect of the intervention on RA patients’ cardio-respiratory fitness (VO_2_ max), PA levels (self-reported and objectively assessed), CVD risk factors (e.g., blood pressure, serological markers), RA disease activity and severity, and motivational processes underlying levels of PA engagement, immediately post-programme and at 6 and 12 month follow-up (Clinical trials registration number: ISRCTN04121489).

It is hypothesised that participants randomly allocated to the multi-component intervention arm will demonstrate more autonomous motivation for PA engagement, as well as higher levels of PA and more positive well-being immediately post intervention, and at 6 and 12 months follow-up, compared to participants who receive the exercise programme without psychological intervention. It is also hypothesised that higher adherence to the exercise programme will be associated with an improvement in cardio-respiratory fitness, quality of life and psychological well-being, as well as experiencing reductions in markers of CVD risk (e.g., blood pressure).

## Methods

Participants with RA were recruited from consecutive rheumatology outpatient clinics at the Dudley Group NHS Foundation Trust in the UK, with recruitment taking place over a 30 month period. Patients with RA, according to the ACR criteria [48], and without co-morbidities prohibiting exercise were recruited. Exclusion criteria were recent joint surgery (in preceding 6 months), fibromyalgia and co-morbidity incompatible with exercise as per ACSM guidelines. Ethical approval was obtained from the Birmingham, East, North and Solihull Research NHS Ethics Committee (protocol number 10/H1206/59).

### Protocol

Patients that satisfied the inclusion criteria were approached by a member of the research team in the waiting room of rheumatology clinics and provided with information about the study. After written consent was obtained, participants were randomised into the intervention or control condition. Randomisation was completed by a third party (Cancer Clinical Trials Unit, University of Birmingham) and stratified based on gender. Participants randomised to the experimental arm were provided with an additional information sheet concerning the content of the psychological intervention (see Table 1), and then re-consented. The two stages of consent were necessary to reduce contamination between the two arms, as providing a complete description of the psychological intervention to participants in both study arms would have jeopardised the internal validity of the study.

**Table 1:** **Psychological intervention content for each contact with participants**

Consultation	Contact	Duration	Behaviour change techniques
Baseline	Face to face	60 mins	• Elicit and acknowledge positive and negative experiences and emotions towards physical activity
			• Identify the patient’s knowledge regarding the benefits associated with increasing physically active behaviour specific to RA; gear discussions of these benefits to what is personally meaningful to the patient
			• Provide additional information requested by the patient
			• Encourage reflection on the links between physically active behaviour and personally meaningful life goals or events
			• Decisional balance
			• Patient centred goal setting
1 month	Telephone	10 mins	• Support attempts to change behaviour
			• Normalize failed attempts to be physically active
			• Problem solve to formulate strategies for enhancing self-efficacy
			• Elicit solutions to PA barriers
			• Revisit goals
2 month	Telephone	10 mins	• Encourage attempts made to be physically active
			• Brainstorm solutions to PA barriers
			• Discuss patient goals for last period of programme
3 month	Face to face	30 mins	• Recognise the internalisation of individual’s PA participation
			• Have patients verbalise feelings towards physical activity
			• Discuss plans to be physically active in the future
			• Information regarding where it is possible to be physically active
5 month	Telephone	10 mins	• Discuss successful and failed attempts to maintain PA behaviour post exercise-programme

All participants were invited to participate in a 3-month exercise programme in a local gym. The cost of the gym membership was covered to remove this known barrier to PA engagement in gym-based activities [49]. Participants completed assessments at 4 time points: baseline (before starting the exercise programme), 3 months (immediately following the completion of the exercise programme), 6 months and 12 months. Each time point consisted of securing 2 different arrays of assessments. Assessments were carried out at the Clinical Research Unit within the Dudley Group NHS Foundation Trust, 7 days apart. During the first assessment appointment (approximately 30 minutes), a fasted blood sample was taken and participants were asked to complete a questionnaire pack (See Table 2). A sub-sample (85%) of participants were provided with an Actigraph accelerometer (GT3X) to be worn for 7 days. Verbal and written instructions were provided detailing how the accelerometer should be worn and a demonstration given. Participants were requested to wear the device for the next 7 days, only removing for showering/bathing.

**Table 2:** **Secondary outcome measures for the RCT**

Outcome	Measure
**Physical activity related**	
Physical activity adherence	Attendance
Physical activity (self-report)	International Physical Activity Questionnaire [50]
Physical activity (objective)	Actigraph GT3X
Autonomy support (fitness instructor)	Health Care Climate Questionnaire [42]
Autonomy support (important others & PA advisor)	Important Other Care Climate Questionnaire [51]
Need satisfaction	Need Satisfaction in Exercise Questionnaire [52]
Motivational regulations	Behavioural Regulations in Exercise Questionnaire-2 [53]
**Health related**	
Acute phase response	Routine laboratory assessments (i.e., C-Reactive Protein)
Disease activity	Disease Activity Score 28 [54]
Quality of life	EQ-5D-3L [55]
Anxiety & depression	Hospital Anxiety and Depressions Scale [56]
Well-being	Subjective Vitality Scale [57]
Fatigue	Multidimensional Assessment of Fatigue Scale [58]
Physical function	Stanford Health Assessment Questionnaire (Kirwan & Reeback) [59]
Pain	McGill Pain Questionnaire [60]
**Economic costing**	
Changes in capability	ICECAP-A [61, 62]
Quality-adjusted life-years	EQ-5D-3L [55, 63]

**Figure 1:**
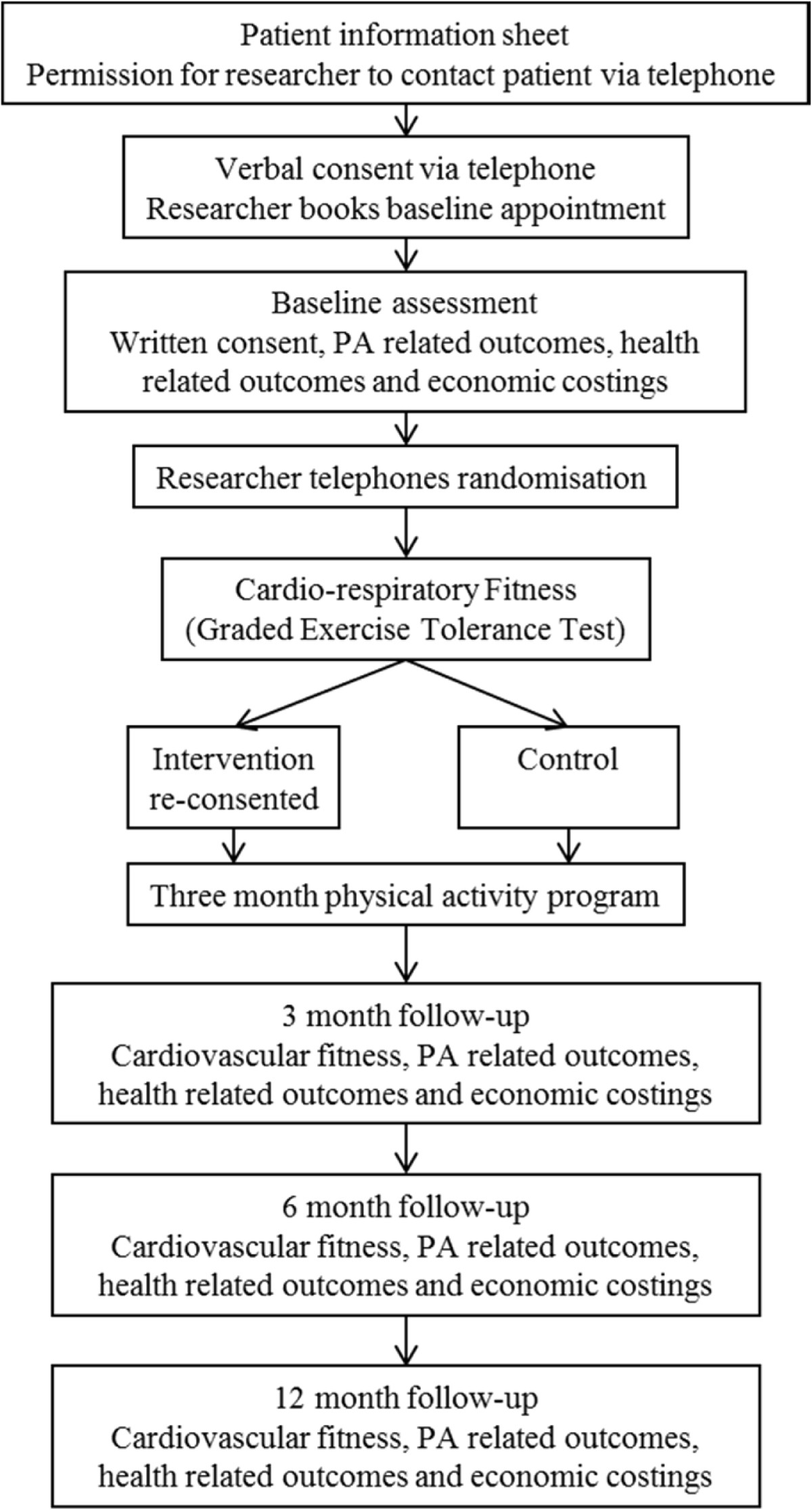
**A flow diagram of the physical activity in rheumatoid arthritis (PARA) study design.**

At the second assessment appointment (taking approximately 50 minutes), an exercise tolerance test (ETT) with electrocardiography (ECG) was conducted by two trained exercise and cardiac physiologists. Assessors were blinded to the intervention arm (i.e., experimental vs. control) to which participants were randomised. Participants returned the accelerometer and completed a self-report measure of PA (i.e., The International Physical Activity Questionnaire; IPAQ) [50] along with a demographic information sheet. See Figure 1 for the study flow diagram.

### Physical activity intervention

All participants, regardless of study arm, participated in a 3-month exercise programme. To avoid contamination, the two arms completed the programmes at two separate gym centres. Both gym centres were similar in terms of facilities, size, available equipment, environment. Gym instructors at both exercise centres received training in how to support patients with RA by the same exercise physiologist with extensive experience in developing exercise programmes appropriate for patients with RA. This training consisted of information about RA in general, how to develop a safe programme for this patient group, as well as the potential physical and psychological barriers RA patients may encounter while participating in an exercise programme.

Following the baseline assessment of cardio-respiratory fitness, an individualised 3-month exercise programme was developed, using all available equipment (treadmills, hand ergometers, etc) and according to the patient’s preference and ability [26]. In line with the American College of Sports Medicine (ACSM) guidelines (2005) for exercise prescription, participants were asked to exercise at 60-75% of their VO_2_ max (indicated by monitored heart rate) for a duration greater than 30 min during each exercise session. All exercise sessions were designed to last approximately 55mins and consisted of10mins warm-up, 30-40mins main session, and 5-10mins cooling down.

Exercisers provided one-to-one supervision to participants during their initial exercise session. This first gym session served as an induction to the exercise equipment and exercise programme. In the case that a participant required further assistance, full supervision was provided until he or she felt familiar and comfortable with the equipment and confident to exercise alone. Thereafter, patients exercised in a semi-supervised setting. Instructors were available to assist if patients required any further support, or in case of emergency. In total, three exercise sessions per week were recommended to all participants; two sessions conducted in the gym environment within the designated exercise centre, and one session at home. Objective attendance records were obtained from both exercise centres. At the end of the 3-month programme, patients were left to their own devices to continue their engagement in PA.

### Psychological intervention

For patients randomised to the experimental arm, the individualised 3-month exercise programme was supplemented by psychological intervention strategies grounded in SDT [64]. Strategies aimed to facilitate the adoption and maintenance of PA, whilst supporting the patients’ autonomous motivation for PA engagement and psychological well-being via one-to-one consultations with a PA behavioural change counsellor [64]. The counsellor had contact with each patient at 4 time points during the 3-month exercise programme (i.e., a 1 hr face to face consultation at programme induction, brief consultations via telephone at 1 and 2 months, and an approximately 30 min face to face consultation at 3 months). As the literature points to the need for a follow up consultation when participants are trying to maintain their PA post-exercise programme [65], a final telephone consultation took place at 5 months (i.e., 2 months following the end of the exercise intervention). This approach is in line with the recommendations of Hillsdon and colleagues [66] regarding the minimal frequency of intervention occasions, and recent work involving SDT-grounded exercise on referral consultations [28]. This protocol also parallels the SDT-based behavioural change interventions employed by Williams and colleagues [40] (smoking cessation/dietary change) and other researchers centred on PA promotion specifically [67].

The counsellor was trained to employ particular strategies and approaches targeting the promotion of more autonomous motivation for PA behaviour change in the participant. For example, during the initial consultation, the counsellor elicited and acknowledged positive and negative experiences and emotions towards PA, provided personally salient information regarding the benefits associated with increasing physically active behaviour specific to RA, and encouraged reflection on the links between physically active behaviour and personally meaningful life goals or events. See Table 1 for details of intervention content.

To reduce the potential that the exercise instructors who supervised the sessions might be creating an environment in the gym which is at odds to the perspective adopted within the one on one consultations, two 1.5 hours information sessions were provided. To create a need supportive exercise environment, exercise instructors at the experimental gym centre received information on the benefits of promoting autonomy (e.g,. offering choice of the types exercises included in his/her programme), providing structure to enhance feelings of competence (e.g., encouraging patients to ask question) and supporting relatedness (e.g., dedicating time and attention to the patient) during exercise sessions with RA patients. Within these sessions, the instructors were encouraged to identify current good practice and generate strategies by which they could be more need supportive when supervising gym activities.

### Outcomes

#### Primary outcome

Cardio-respiratory fitness (CRF) was the primary outcome and was assessed using a graded exercise tolerance test, either on a treadmill (HP Cosmos Mercury, Nussdorf-Traunstein, Germany) or an exercise bike (Lode, The Netherlands), depending on the patient’s ability and preference. Based on guidelines from the American Heart Association, an individualised test protocol was utilised to assess CRF [68]. The testing initiated in most cases at 2mph and 1% inclination. The speed increased by 0.5mph every 1 minute until 4.0mph followed, thereafter, by inclination increases every 30 seconds by 1%. In all cases, the aim was to achieve exhaustion within 7-12 minutes. CRF was determined using a calibrated breath-by-breath system (Metalyzer 3B, CORTEX Biophysik GmbH, Leipzig, Germany) allowing continuous measurement of oxygen uptake (VO_2_), and lung ventilation, determined once every 30 seconds. Additionally, blood pressure was monitored and heart rate and function were continuously assessed via a 12 lead electrocardiogram.

### Secondary outcomes

#### CVD risk factors

Resting blood pressure and serological risk factors, including full blood count, serum biochemistry, Erythrocyte Segmentation Rate (ESR), high-sensitivity C-Reactive Protein (hsCRP), blood glucose, lipids and thrombotic variables were assessed using routine laboratory procedures. Internal quality controls on all analysers are carried out daily, and external quality controls fortnightly, utilising the Welsh External Quality Assurance Screen (WEQAS). On the basis of these individual CVD risk factors, the 10-year CVD event probability was calculated using the latest (2006) version of the Joint British Societies’ risk calculator [69].

### Physical activity behaviours

(a) Adherence to intervention: assessment of attendance at weekly exercise sessions, (b) self reported PA was be assessed using the International Physical Activity Questionnaire (IPAQ) [50]. The IPAQ measures the level of PA across four domains; leisure time PA, domestic and gardening (yard) activities, work - related PA, and transport - related PA, and (c) objective PA assessed by Actigraph accelerometers (GT3X) over a 7 day period in a sub-sample from each arm (experimental, N = 50, control, N = 48).

### Health and well being indicators

(a) Health related quality of life was measured using the EuroQol, five dimensions, three levels (EQ-5D-3L) [55]. The EQ-5D-3L determines self-assessed problems across five items of mobility, self-care, usual activities, pain/discomfort and anxiety/depression. Each item has three levels of severity: ‘no problems’ , ‘some problems’ and ‘severe problems’. The resulting health states were valued using the UK tariff [63]. (b) anxiety and depression were assessed using the Hospital Anxiety and Depression Status (HADS) questionnaire [56]. The HADS consists of 14 items (7 each for anxiety and depression) rated on various four-point scoring systems (c) positive mental health was assessed via the Subjective Vitality Scale [57], (d) capability wellbeing was assessed using the ICECAP-A capability index for adults [61]. The ICECAP-A consists of five items of Attachment, Stability, Achievement, Enjoyment and Autonomy. The resulting capability states were valued using the UK tariff [62].

### Motivation and processes of change

(a) Perceptions of autonomy support, perceived efficacy/competence, autonomy, social connectedness with respect to PA engagement, and motivational regulations for exercise were determined using existing scales which were validated for use in the case of RA patients.

Perceptions of autonomy support were assessed using an adapted version of the Health Care Climate Questionnaire (HCCQ) [42]. This 15 item scale was designed to measure the degree of autonomy support (vs. controllingness) perceived to be provided by the PA counsellor and their exercise instructor (e.g., I feel my PA advisor has provided me with choices and options). Answers were responded to on a 7-point Likert scale ranging from 1 (strongly disagree) to 7 (strongly agree).

Perceived competence, autonomy and social connectedness were assessed via participants responses to the 18-item Psychological Need Satisfaction in Exercise Scale (PNES) [52]. This measure consists of three, six item subscales, i.e., perceived competence (e.g., I am confident that I can do even the most challenging exercises), autonomy (e.g., I feel free to exercise in my own way) and relatedness (e.g., I feel close to my exercise companions who appreciate how difficult exercise can be). Answers are rated on a 6-point Likert scale ranging from 1 (false) to 6 (true).

Motivation regulations were measured using an adapted version of the Behavioural Regulation in Exercise Questionnaire (BREQ-2) [53]. This 19 item questionnaire assesses behavioural regulation in the context of exercise. Specifically, participants are asked to respond to items reflecting intrinsic motivation (e.g., I exercise because it is fun), identified regulation (e.g., I value the benefits of exercise), introjected regulation (I feel guilty when I don’t exercise), external regulation (e.g., I exercise because other people say I should), and amotivation (e.g. I don’t see the point of exercising), rating their agreement with each statement on a 4-point Likert scale ranging from 0 (not true for me) to 4 (very true for me).

### RA activity and severity

Disease Activity was measured using the DAS28 [54]. This is a composite assessment consisting of the patient’s appraisal of overall health during the last week on a visual analogue scale, a 28 joint count and the current ESR. Acute phase response (i.e., CRP) was also measured using routine laboratory assessments. Physical function was assessed using the Anglicised version of the 40-item Stanford Health Assessment Questionnaire (HAQ) [59]. Patients rated their ability (over the past week) to carry out 20 activities within eight aspects of daily living (dressing/grooming, rising, eating, walking, hygiene, reach grip and errands/tasks) on a four-point scale from ‘without any difficulty’ to ‘unable to do’. For each aspect patients also responded whether they receive assistance from people or use specific devices. Pain was assessed using the McGill Pain Questionnaire [60]. Patients are asked to rate 11 sensory descriptors (e.g., shooting, aching, splitting) and 4 affective descriptors (e.g., tiring, sickening) on an intensity scale ranging from 0 (none) to 3 (severe). Fatigue was measured using the Multidimensional Assessment of Fatigue scale (MAF) [58]. This 16-item scale measures four dimensions of fatigue: severity, distress, degree of interference in activities of daily living, and timing. Fourteen items contain numerical rating scales and two items have multiple-choice responses. Patients will be asked to reflect on fatigue patterns for the past week.

### Economic analysis

The economic evaluation took the form of two cost-effectiveness analyses from the perspective of the National Health Service (NHS). In the first, costs were related to the changes in quality-adjusted life-years (QALYs) developed using information collected from EQ-5D-3L [55, 63]; in the second, costs were related to changes in capability using the ICECAP-A capability index for adults [61, 62]. A secondary analysis took the form of a cost-consequences analysis reporting all trial outcomes and costs from the perspective of both NHS and patients.

### Power calculations

Our power calculations (nQuery Advisor v 6.0, Statistical Solutions, MA, USA) were based on the primary outcome CRF and indicated that we needed 50 participants per group. This is sufficient to detect an estimated mean difference in CRF of 2 ml/kg/min (SD_diff._ = 3) at 1 year between the 2 groups (power = 80%; alpha = .05), allowing for 20% drop out rate. However, groups were compared on an intention to treat basis, thus, even patients that drop-out were included in the analyses.

### Statistical analysis strategy

Multilevel regression analyses were used to test the effect of the intervention on our outcome measures in comparison with the standard exercise provision arm, adjusting follow up scores for baseline scores (where available) and key baseline characteristics (e.g., age/sex, disease duration). Structural equation modelling was used to test a theory-based process model examining social psychological and motivation-related antecedents and mediators of change in targeted outcomes.

## Discussion

This paper details the rationale and protocol for conducting an RCT investigating the effect of participation in two exercise programmes which were customised for people with RA, exercise only versus exercise with an additional psychological intervention that aimed to enhance the adoption and maintenance of PA, autonomous motivation for PA engagement, and associated indicators of health, QOL, and psychological well being. The psychological intervention was grounded in SDT [64], a contemporary motivation theory that provides guidance regarding key social environmental factors that can be manipulated to support motivational processes conducive to positive health behaviour change and optimal functioning.

## Authors’ original submitted files for images

Below are the links to the authors’ original submitted files for images. Authors’ original file for figure 1

## Abbreviations

PA: Physical activity
RA: Rheumatoid arthritis
CVD: Cardiovascular disease
CRF: Cardio-respiratory fitness
ETT: Exercise tolerance test
SDT: Self-determination theory
RCT: Randomised control trial
NHS: National health service
QALYs: Quality adjusted life years
EQ-5D-3L: EuroQol, five dimensions, three levels
ICECAP-A: Investigating choice experiments capability index for adults.
