# Supplementary figures and images for: Fostering autonomous motivation, physical activity and cardiorespiratory fitness in rheumatoid arthritis: protocol and rationale for a randomised control trial
Source: BMC Musculoskelet Disord. 2014 Dec 19;15:445. doi: 10.1186/1471-2474-15-445 (PMC4320601; doi:10.1186/1471-2474-15-445)

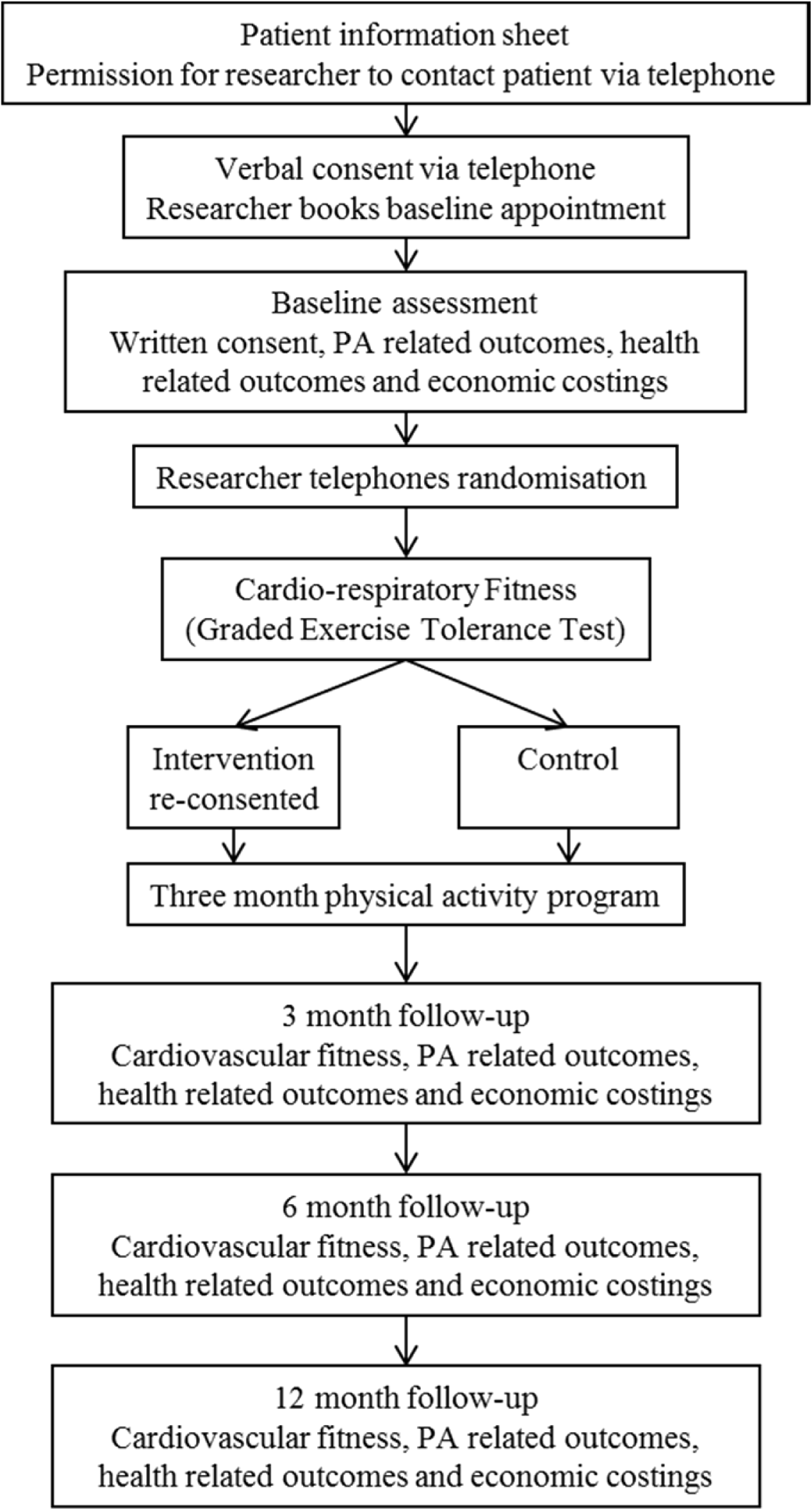

Supplement: Supplementary file 1 — Authors’ original file for figure 1 [file 12891_2014_2392_MOESM1_ESM.tif]
